# Supplementary material for: Acidic graphene organocatalyst for the superior transformation of wastes into high-added-value chemicals
Source: Nat Commun. 2023 Mar 13;14:1373. doi: 10.1038/s41467-023-36602-0 (PMC10011376; doi:10.1038/s41467-023-36602-0)
Supplement: Supplementary file 1 — Supplementary Information [file 41467_2023_36602_MOESM1_ESM.pdf]

Supplementary Information

Acidic graphene organocatalyst for the superior transformation of wastes into high-added-value chemicals

Aby Cheruvathoor Poulose,[1] Miroslav Medveď,[1,2] Vasudeva Rao Bakuru,[3] Akashdeep Sharma,[4] Deepika Singh,[5] Suresh Babu Kalidindi,[6] Hugo Bares,[1,#] Michal Otyepka,[1,7] Kolleboyina Jayaramulu,[4,*] Aristides Bakandritsos,[1,8,*] Radek Zbořil[1,8,*]

[1]Regional Centre of Advanced Technologies and Materials, Czech Advanced Technology and Research Institute (CATRIN), Palacký University in Olomouc, Šlechtitelů 27, 783 71, Olomouc, Czech Republic

[2] Department of Chemistry, Faculty of Natural Sciences, Matej Bel University, Tajovského 40, 974 01 Banská Bystrica, Slovak Republic

[3] Materials Science and Catalysis Division, Poornaprajna Institute of Scientific Research, Bangalore Rural, India

[4]Department of Chemistry, Indian Institute of Technology Jammu, Nagrota Bypass Road, Jammu, Jammu and Kashmir 181221, India

[5]CSIR-Indian Institute of Integrative Medicine Jammu, Canal Road, Jammu, Jammu and Kashmir, 180001 India

[6] Department of Inorganic and Analytical Chemistry, School of Chemistry, Andhra University, Visakhapatnam, India

[7] IT4Innovations, VŠB - Technical University of Ostrava, 17. listopadu 2172/15, Ostrava-Poruba 70800, Czech Republic

[8] Nanotechnology Centre, Centre of Energy and Environmental Technologies, VŠB–Technical University of Ostrava, 17. listopadu 2172/15, Poruba, 708 00 Ostrava, Czech Republic

[#]current address: Lepty, 14 avenue Pey-Berland, 33600 Pessac, France

* Corresponding authors E-mail addresses: jayaramulu.kolleboyina@iitjammu.ac.in; a.bakandritsos@upol.cz; radek.zboril@upol.cz

Supplementary Discussion

Computational details

To gain mechanistic insights into glycerol acetalization reaction over the graphene-aminosulfonic acid (G-ASA) catalyst, the binding characteristics of reagents and individual reaction steps (Figure 4) were analyzed by performing density functional theory (DFT) calculations. The ground state (GS) structures of all the investigated species were optimized by the ωB97X-D method[1] using the 6-31+G(d) basis set[2]. The solvent effects were included by applying the universal continuum solvation model based on solute electron density (SMD)[3] All calculations were performed with the Gaussian16 program[4].

Whereas the structures of small and medium-size systems (glycerol, acetone, H_2O), as well as the parent model structures (Supplementary Fig. 5 and 6), were fully relaxed in geometry optimizations, to reduce the computational costs, the structures involving both G-ASA and molecular reagents and corresponding relaxed scans were obtained by constrained geometry optimizations relaxing only the region actively participating in a particular reaction, i.e., the sulfonic group and the molecular species. Therefore, the reaction pathways are described in terms of the electronic reaction energies.

In the first step, a conformation analysis of G-ASA and its protonated form using two-side substituted models derived from coronene (Supplementary Fig. 5) revealed that zwitterion structures (F, G) are favorable in acetone solution. In the case of the canonical form, structures with intramolecular hydrogen bonding (i.e., G-SO_3H…NHRR', structure D) are preferred. In the acidic environment, both the sulfonic and amino groups are assumed to be protonated (H). For further analysis of the reaction mechanism, the carbon lattice was extended to an ovalene-like structure to avoid potential artifactual interactions between the molecular reagents and edges of the substrate. Also, the protonated taurine moiety on the bottom side of the lattice was substituted with an ammonium group (Supplementary Fig. 6A), as it was not expected to have a significant impact on the reaction. Such a structure was fully re-optimized (including the frequency analysis) and used for the analysis of individual reaction steps displayed in Figure 4 as well as for a comparison with its alkyl chain analog (Supplementary Fig. 15). For each reaction step, a relaxed scan was performed using first rather coarse steps (typically 0.2 Å) along the reaction coordinate (i.e., selected interatomic distance), which was then refined (to ca. 0.05-0.10 Å) in the critical region(s) to estimate the reaction barriers. The final structures as obtained by the relaxed (but constrained as described above) scans were re-optimized to determine the stable arrangements of reaction intermediates and products.

It should be emphasized that the modeled catalytic reaction takes place in highly acidic environment, which implies the possibility of proton transfer events, in which the environment can be involved. To estimate the activation barriers, we performed a series of back and forward (partially relaxed) scans starting from the reactants and products (or intermediates) in each reaction step along a carefully chosen reaction coordinate. In such an approach, it was assumed that the structure changed its protonated state (i.e., the proton transfer occurs) if the potential energy curve became lower along the particular scan. For example, during the formation of an adduct A without the catalyst (Supplementary Fig. 7), the formation of a C–O bond between glycerol was accompanied by a proton transfer from glycerol to the oxygen atom of acetone, which could however be assisted by the acidic environment. Therefore, the barrier shown in Supplementary Fig. 7a was estimated from back and forward scans as shown in Supplementary Fig. 7b. It should also be underlined that the choice of the internal reaction coordinates was chemically well founded, because it could be presumed that the formation of adducts (steps 1-3 in Figure 4) involved an attack of one of the oxygen atoms of glycerol on the carbonyl group of acetone and also the formation of cyclic products (steps 4-6 in Figure 4) required approaching specific atoms.

Supplementary Tables

Supplementary Table 1. Comparisons of previous catalysts with G-ASA for glycerol acetalization.

		Catalyst	Temperature (°C)	Glycerol:acetone molar ratio	Catalyst mass loading% (w.r.t. glycerol)	Reaction time (h)	Glycerol conversion (%)	Solketal selectivity (%)	Acid sites (mmol g^{-1})	Specific productivity[a] (mmol g^{-1} h^{-1})	TOF[b] (h^{-1})	Reference
**Lewis acid**	a	Hf-TUD	80	1:2	3	6	52	100		39		[5]Green Chem., 2012, 14, 1611–1619.
	b	MO_3/SnO_2	25	1:1	5	1.5	71	96		103		[6]Green Chem., 2013, 15, 478–489.
	c	SO_4^{2-}/SnO_2	25	1: 1	5	4	95	96		52		[7]Catal. Sci. Technol., 2014, 4, 803–813.
	d	$NbO_2(OH)$	70	1:2	47	1	65	95		33		[8]ChemCatChem, 2014, 6, 2961–2969.
	e	NbO_2O_5	70	1:1.5	6.4	6	80	92		23		[9]Catal. Sci. Technol., 2012, 2, 1173–1179.
**Brønsted acid**	α	Carbon-SO_3H	25	1:4	5	4	91	98	5.45	50	9	[10]Catal. Sci. Technol., 2020, 10, 4827
	β	Activated carbon–SO_3H	25	1:4	2.7	6	97	96	6.52	65	10	[11]Catal. Sci. Technol., 2014, 4, 2293–2301.
	γ	Al-TFSO_3H-SiO_2	25	1:4	10	4	86	100	1.55	23	15	[12]Catal. Sci. Technol., 2015, 5, 2427–2440.
	δ	SiO_2-SO_3H	70	1:6	5	0.5	80	---	1	347	18	[13]Green Chem., 2010, 12, 899–907.
	ε	Polymer-SO_3H	60	1:5	8	4	97	100	0.65	33	52	[14]Energy Fuels, 2018, 32, 12567–12576.
	ζ	Acidic carbon	25	1:4	3	4	80	95	1.2	72	57	[15]Fuel, 2016, 181, 46–54.
	η	p-Tol-SO_3H	25	1:4	1 mol%	1.5	65	98	—	251	42	[16]Ind. Eng. Chem. Res. 2013, 52, 47, 16709–16713
	θ	H_2SO_4	25	1:4	1 mol%	1.5	59	97	—	400	38	[16]Ind. Eng. Chem. Res. 2013, 52, 47, 16709–16713
**#1**		**G-ASA**	**25**	**1:4**	**0.1**	**1**	**69.2**	**89.3**	**3.87**	**7508**	**1735**	**This work**
**#2**		(ASA=taurine**;**	**25**	**1:4**	**0.25**	**1**	**80.3**	**92.6**		**3485**	**834**	
**#3**		2-aminoethanesulfonic acid)	**25**	**1:4**	**0.5**	**1**	**96.3**	**96.8**		**2094**	**524**	

[a]Specific productivity (mmol g^{-1} h^{-1}) = mmoles of glycerol converted per gram of catalyst per hour
[b]Turnover frequency (h^{-1}) = mmoles of solketal produced per mmol of acid sites per hour

Supplementary Table 2. The relative electronic and standard Gibbs energies (kcal/mol, T = 298.15 K) of the closed form and the TS with respect to the open form of protonated adducts A-H$^+$ and B-H$^+$ calculated at the ωB97XD/6-31+G(d)/SMD level of theory. Note: The TS structures were fully optimized starting from the maxima obtained by relaxed scans displayed in Figure S11.

	Adduct A-H$^+$		Adduct B-H$^+$	
	ΔE	$\Delta G°$	ΔE	$\Delta G°$
Open form	0.0	0.0	0.0	0.0
Closed form	3.8	7.0	4.0	7.4
TS	5.2	7.2	4.4	7.2

Acid-base equilibria

A: G-NH_2^+-R-SO_3H (ace) → G-NH_2^+-R-SO_3^- (ace) + H^+(ace)

B: G-CH_2-R-SO_3H (ace) → G-CH_2-R-SO_3^- (ace) + H^+(ace)

Evaluation of relative pK_a:

Supplementary Table 3. The standard Gibbs energies (kcal/mol, T = 298.15 K) and corresponding ΔpK_a of G-ASA (A) and G-$(CH_2)_3$-sulfonic acid in acetone (see structures in Figure SX2) calculated at the ωB97XD/6-31+G(d)/SMD level of theory.

Acid	$G^{\circ}_{sol}(\text{X}) - G^{\circ}_{sol}(\text{X-H}^+)$
G-NH_2^+-R-SO_3H (A)	262.0
G-CH_2-R-SO_3H (B)	272.7
$\Delta\Delta G°$	10.7
ΔpK_a (B – A)	**7.9**

Comment: The protonated G-ASA is notably more acidic compared to analogous G-alkylsulfonic acid, which is probably one of the key factors of its higher catalytic activity in the solketal synthesis.

Supplementary Table 4. ICPMS data for the G-CN and G-ASA.

Element	Result (μg/g)		
	G-CN	G-ASA (1)	G-ASA (2)
Cr	18.4	2.9	1.2
Mn	12.7	0.5	0.9
Fe	207.4	12.4	19.4
Co	LTQL[#]	LTQL	LTQL
Ni	54.8	6.8	9.9
Cu	11.7	5	13.6
Pd	LTQL	LTQL	LTQL
Pt	LTQL	LTQL	LTQL

#Lower than quantification limit (LTQL)

Supplementary Table 5. Comparison with fatty acid esterification reaction using previously reported heterogeneous catalysts

	Catalyst	Wt %	Alcohol:Oil	T (°C)	t (h)	Yield (%)	Reference
1	$C\text{-}SO_3H$	7	10:1	80	4	100	[17]Nature 2005, 438, 178
2	$CMK\text{-}5\text{-}SO_3H$	3.3	10:1	70	6	80	[18]Chemistry of Materials 2007, 19, 2395
3	$PDVB\text{-}SO_3CF_3$	6	92:1	65	16	99.9	[19]Journal of American Chemical Society 2012, 134, 16948
4	$aC\text{-}SO_3H$	17	25:1	95	1	62	[20]ACS Catalysis 2012, 2, 1296
5	PSi-SO3H	12.5	5:1	80	6	89.7	[21]Nature Communication 2014, 5, 3170
6	$C\text{-}SO_3H\text{-}C18$	24.5	5:1	70	4	87.3	[22]Journal of Material Chemistry A 2014, 2, 11195
7	$HS/C\text{-}SO_3H$	3.5	10:1	80	5	96.9	[23]ACS Applied Materials & Interfaces 2015, 7, 26767
8	Sulfur-rich GO	0.1g	22:1	65	8	92	[24]ChemSusChem 2017, 10, 3352
9	$Zr_{1.0}Fe_{1.5}\text{-}SA\text{-}SO_3H$	9	12:1	90	4	99.5	[25]Renewable Energy 2019, 139, 688
10	$MF\text{-}SO_3H$	4	8:1	70	2	95.9	[26]Fuel, 2020, 266, 117149
**11**	**G-ASA**	**7**	**20:1**	**60**	**4**	**100**	**This work**

Supplementary Figures

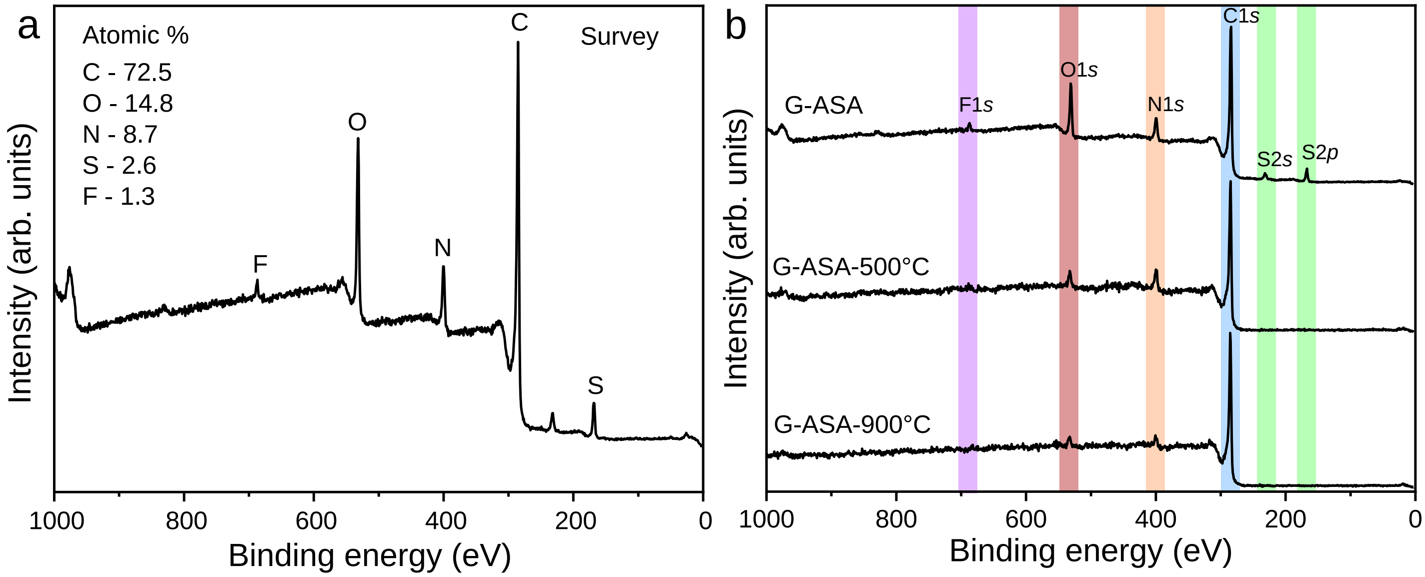

Supplementary Fig. 1. **XPS survey spectrum of G-ASA** (**a**) after synthesis and purifications and (**b**) after thermal treatment under N_2 atmosphere at 500 and 900°C.

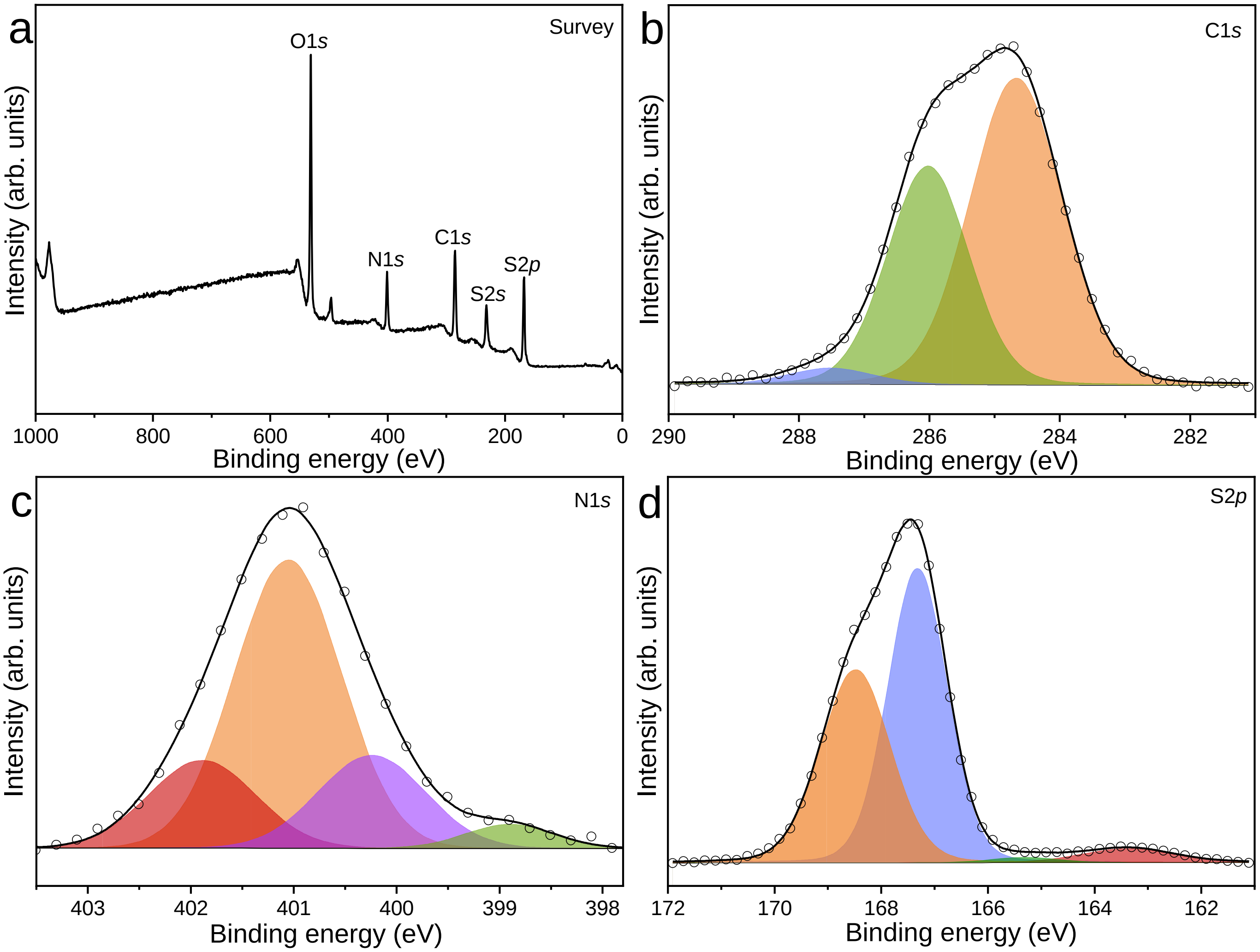

Supplementary Fig. 2. **XPS spectrum of pure taurine. (a)** survey, and core-level spectra for **(b)** C 1s, **(c)** N 1s, and (**d**) S 2p. The very small component in panel c, below 399 eV is attributed to some of the taurine's amino groups interacting with the metallic Si XPS holder. The same is observed in panel d regarding the S component at low eVs, ca. 163.2 eV. The rest of the three components in the N 1s spectrum correspond (as binding affinities increase) to –C–NH_2, hydrogen-bonded –C–N(H_2)···H, and protonated –C–NH_3^+.

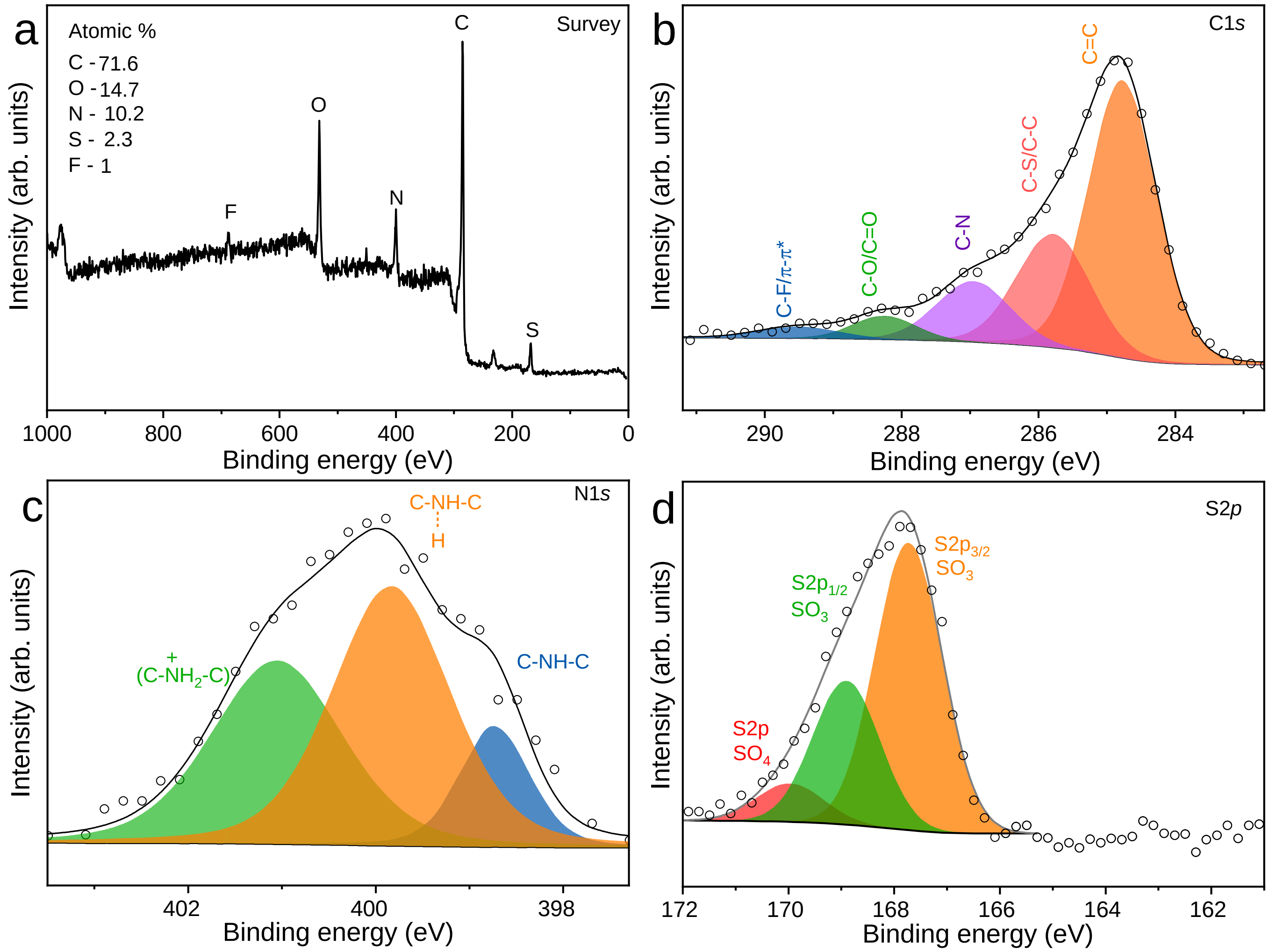

Supplementary Fig. 3. XPS spectrum of G-ASA after 3 reactions. (**a**) survey, and core-level spectra for (**b**) C 1s, (c) N 1s, and (**d**) S 2p.

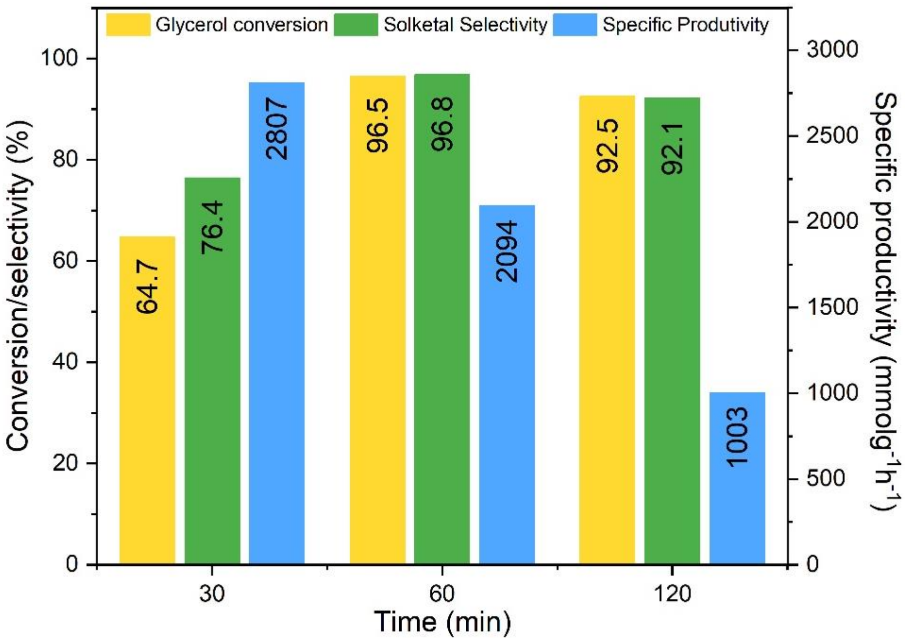

Supplementary Fig. 4. Time resolved study of the glycerol acetylation reaction. Reaction conditions: glycerol = 1.0 g (10.85 mmol), acetone = 2.52 g, (43.38 mmol) Glycerol: acetone = 1:4, catalyst loading=0.5 wt% (with respect to glycerol), the reaction carried out individually at different time periods at room temperature.

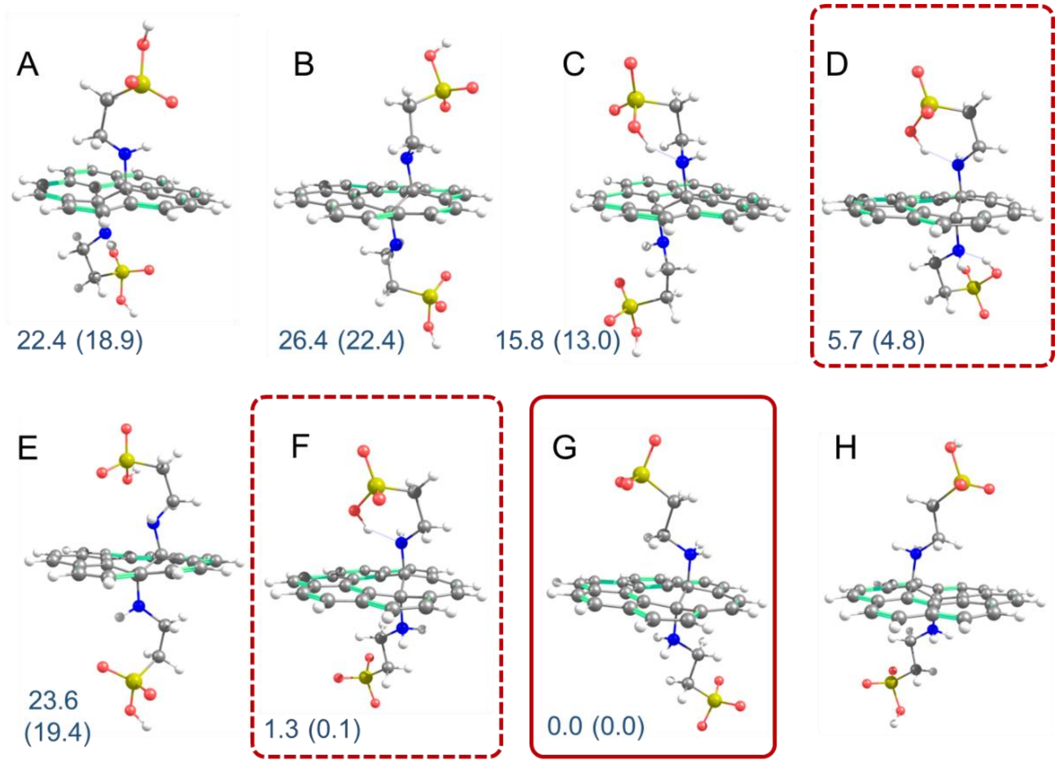

Supplementary Fig. 5. **Model structures of G-ASA.** Conformational analysis of G-ASA (A-G) and its protonated form (H) in acetone. Relative electronic energies and standard Gibbs energies (reported in parentheses, T = 298.15 K) for fully optimized structures were obtained at the ωB97X-D/6-31+G(d)/SMD level of theory. All values are given in kcal/mol.

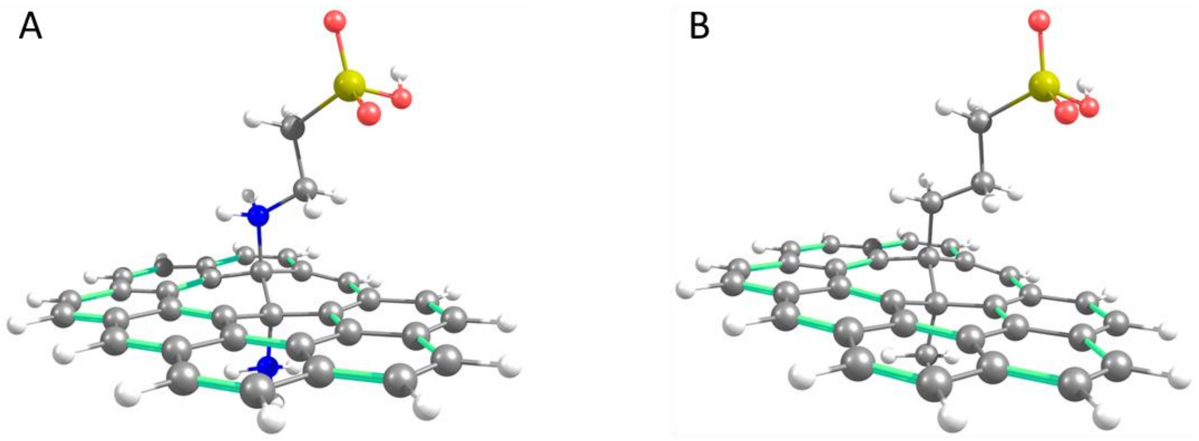

Supplementary Fig. 6. **Simplified model structures of G-ASA.** Model structures of protonated G-ASA (**A**) and G-$(CH_2)_3$-sulfonic acid (**B**) in acetone optimized at the ωB97X-D/6-31+G(d)/SMD level of theory used to unravel the reaction mechanism.

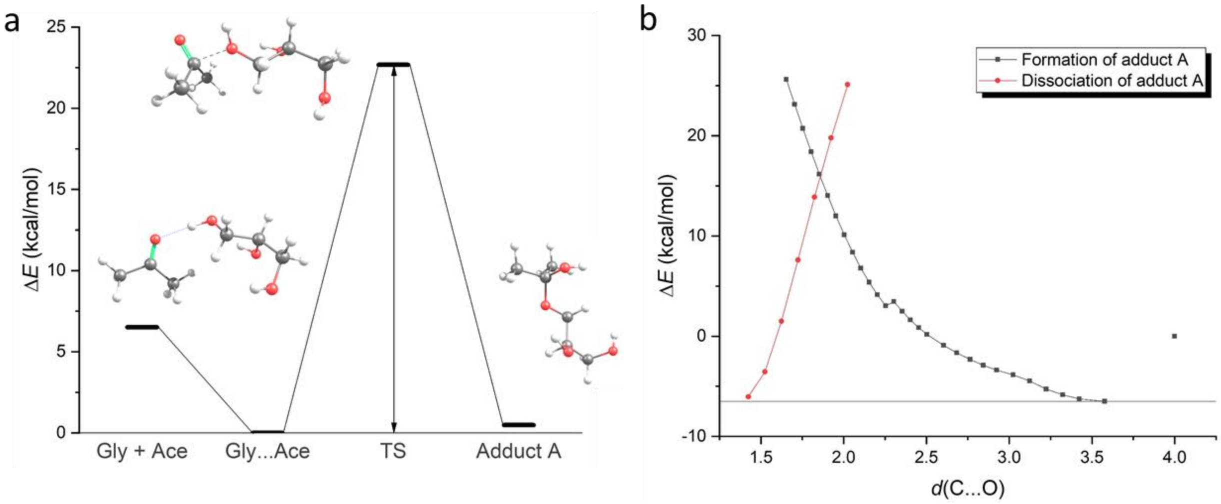

Supplementary Fig. 7. **Formation of adduct A without catalyst.** Energy diagram (**a**) and the corresponding relaxed scan along the C(ace)···O(gly) coordinate (**b**) of the formation of an adduct from acetone and glycerol in acetone with the catalyst. ωB97X-D/6-31+G(d)/SMD(solvent=acetone).

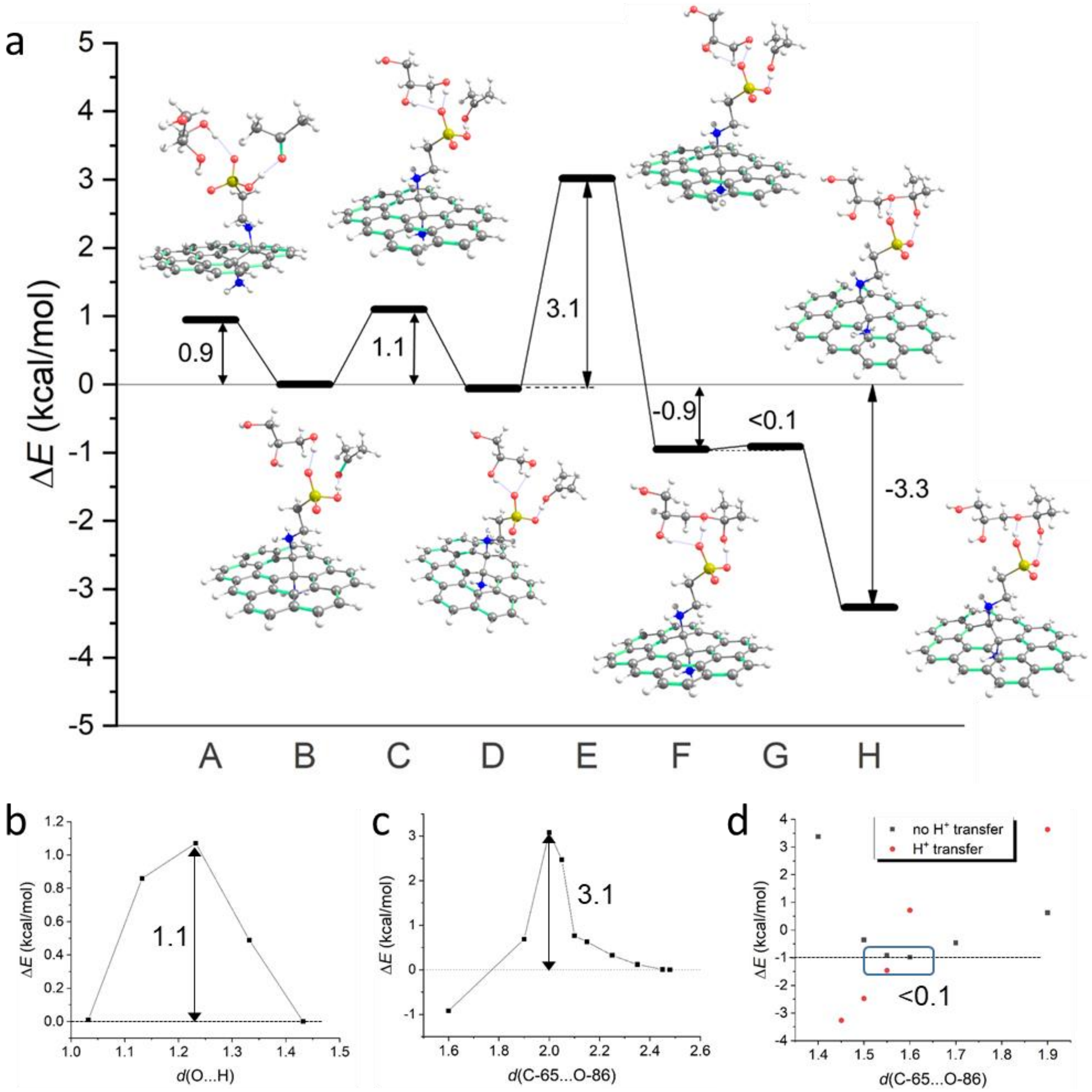

Supplementary Fig. 8. **Formation of adduct A with catalyst.** (a) Energy diagram (in kcal/mol) of the first phase of the catalyzed reaction, i.e. the formation of an adduct A (steps 2a and 3a in Figure 5) along the C-65(acetone)···O-86(gly) coordinate (see Figure S8). (b) Relaxed scan along the O...H coordinate corresponding to a proton transfer from sulfonic group to an oxygen atom of acetone (step B → C → D in panel a). (c) Relaxed scan along C-65(acetone)···O-86(gly) coordinate (step D → E → F in panel a). (d) Relaxed scan along the C-65(acetone)···O-86(gly) coordinate (step F → G → H in panel a); the black and red points correspond to structures with the hydrogen atom bonded to the adduct (specifically to an etheric oxygen) and sulfonic group, respectively, indicating that the proton transfer is practically barrierless and occurs in the vicinity of the F minimum. Computational level: ωB97X-D/6-31+G(d)/SMD(solvent=acetone).

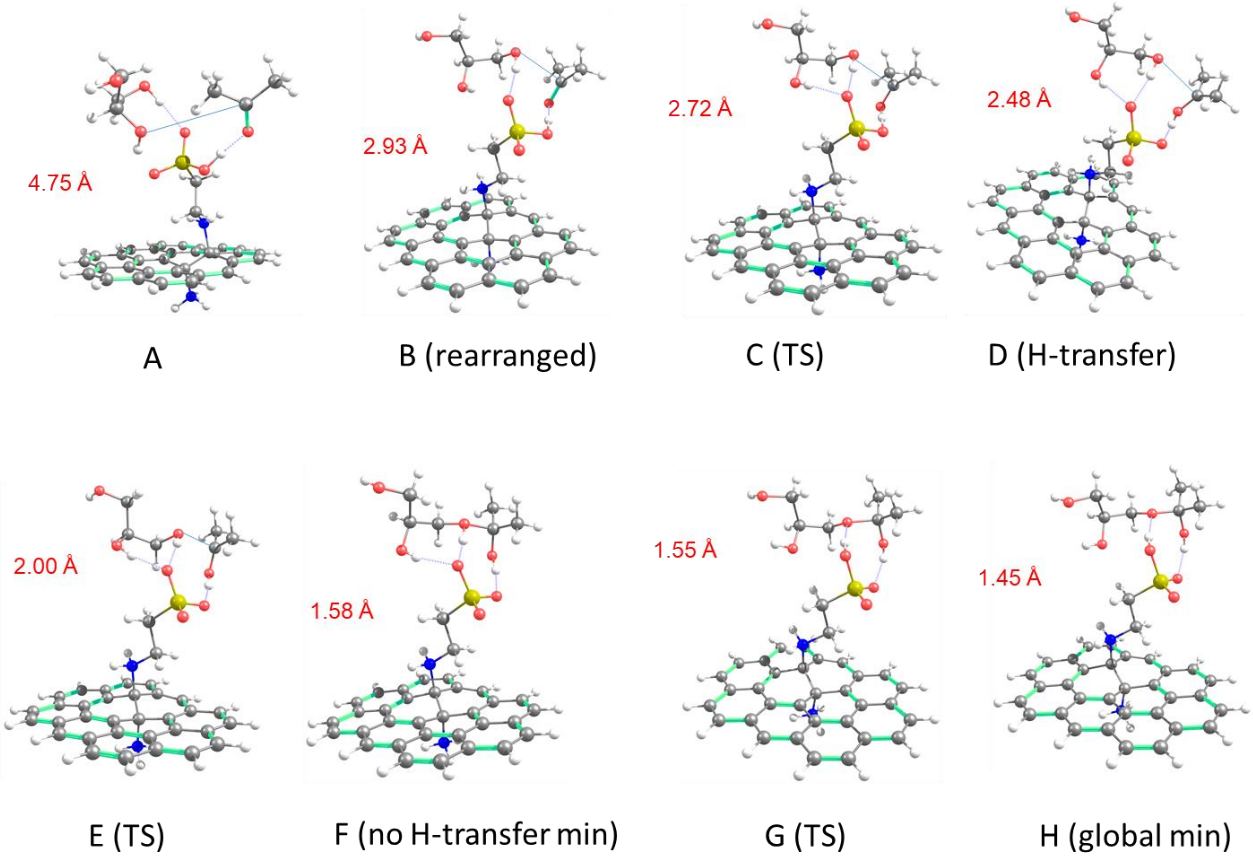

Supplementary Fig. 9. **Key structures of the formation of adduct A.** The structures involved in the formation of adduct A (structure H) from acetone and glycerol in acetone with the catalyst were obtained by a relaxed scan along the C(ace)···O(gly) coordinate. Computational level: ωB97X-D/6-31+G(d)/SMD(solvent=acetone).

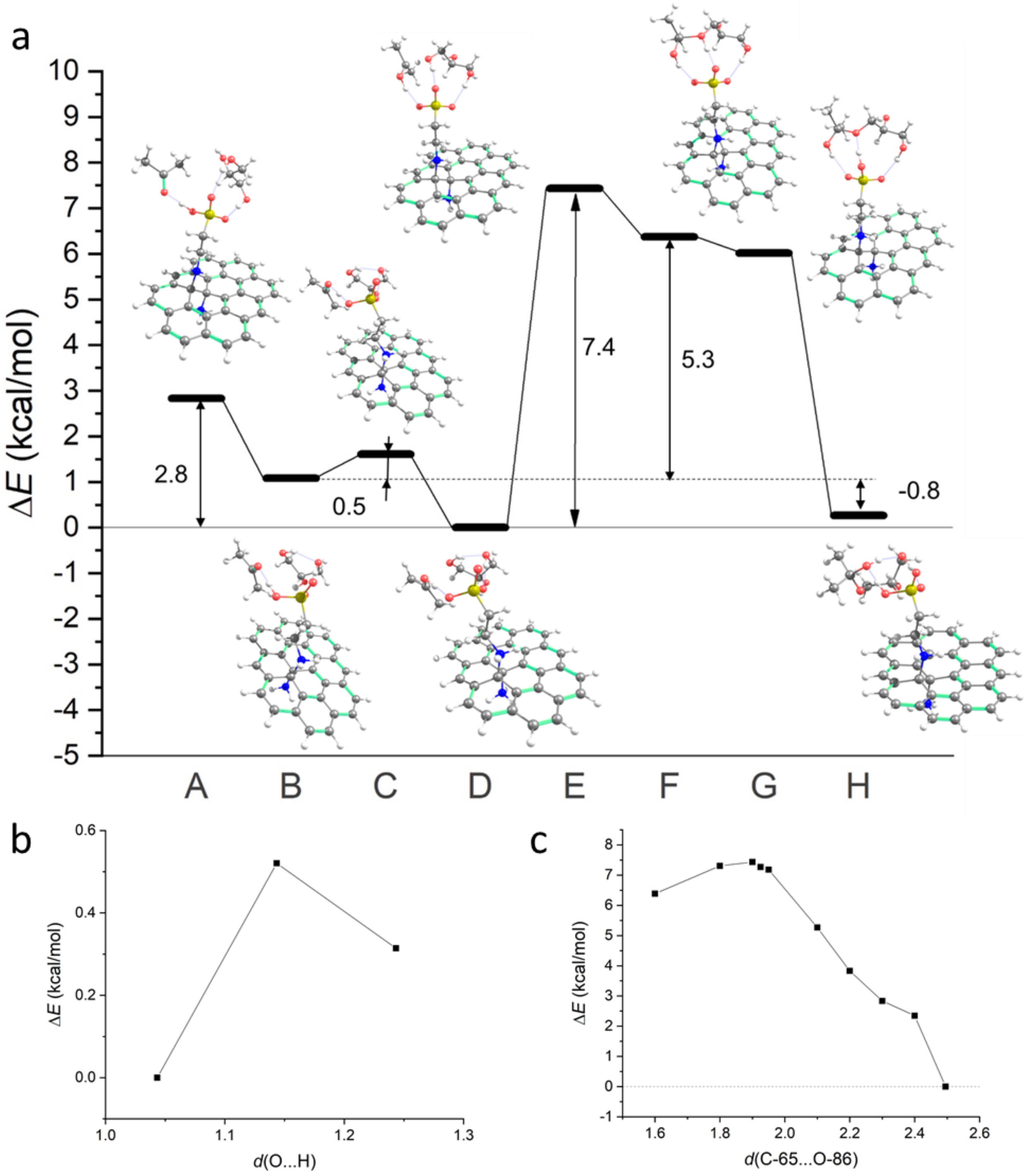

Supplementary Fig. 10. **Formation of adduct B with catalyst.** (a) Energy diagram (in kcal/mol) of the first phase of the catalyzed reaction, i.e. the formation of an adduct B (steps 2b and 3b in Figure 5) along the C(acetone)···O(gly) coordinate (see Figure SX7). (b) Relaxed scan along the O...H coordinate corresponding to a proton transfer from sulfonic group to an oxygen atom of acetone (step B → C → D in panel a). (c) Relaxed scan along the C-65(acetone)···O-86(gly) coordinate (step D → E → F in panel a). Computational level: ωB97X-D/6-31+G(d)/SMD(solvent=acetone).

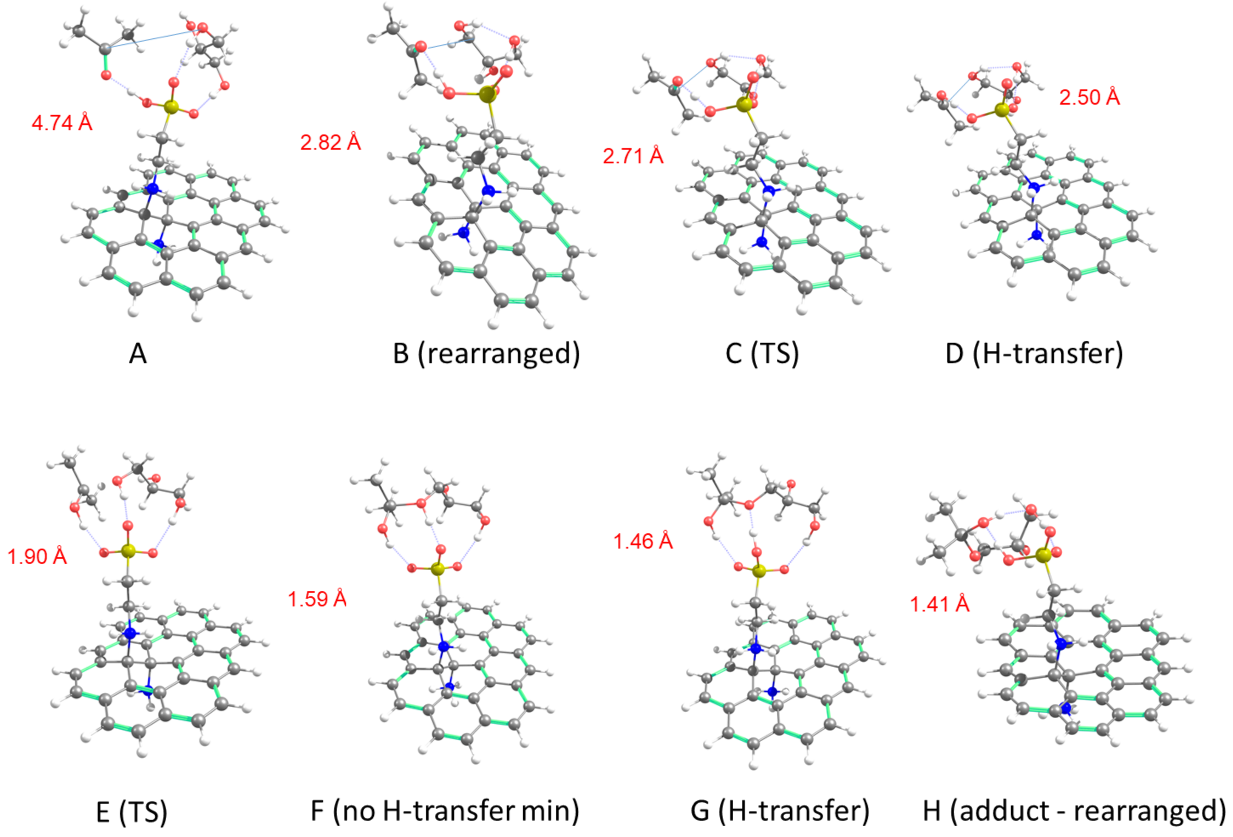

Supplementary Fig. 11. Key structures of the formation of an adduct B. The structures involved in the formation of an adduct B from acetone and glycerol in acetone with the catalyst were obtained by a relaxed scan along the C(ace)···O(gly) coordinate. Computational level: ωB97X-D/6-31+G(d)/SMD(solvent=acetone).

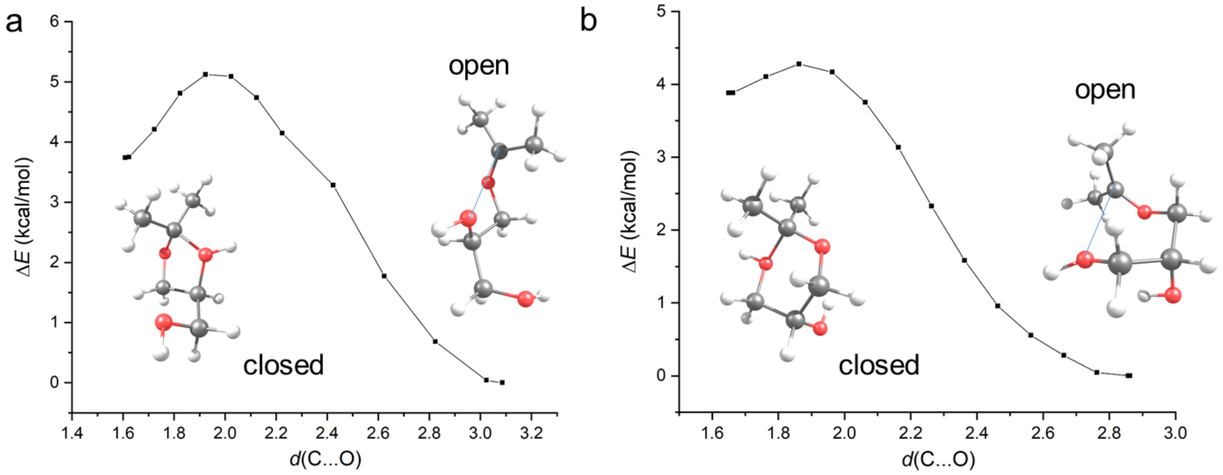

Supplementary Fig. 12. Cyclization of protonated adducts. Relaxed scans for the cyclization of protonated adducts A-H^+ (**a**) and B-H^+ (**b**) along the marked C···O coordinate led to protonated solketal and acetal, respectively. Computational level: ωB97X-D/6-31+G(d)/SMD(solvent=acetone).

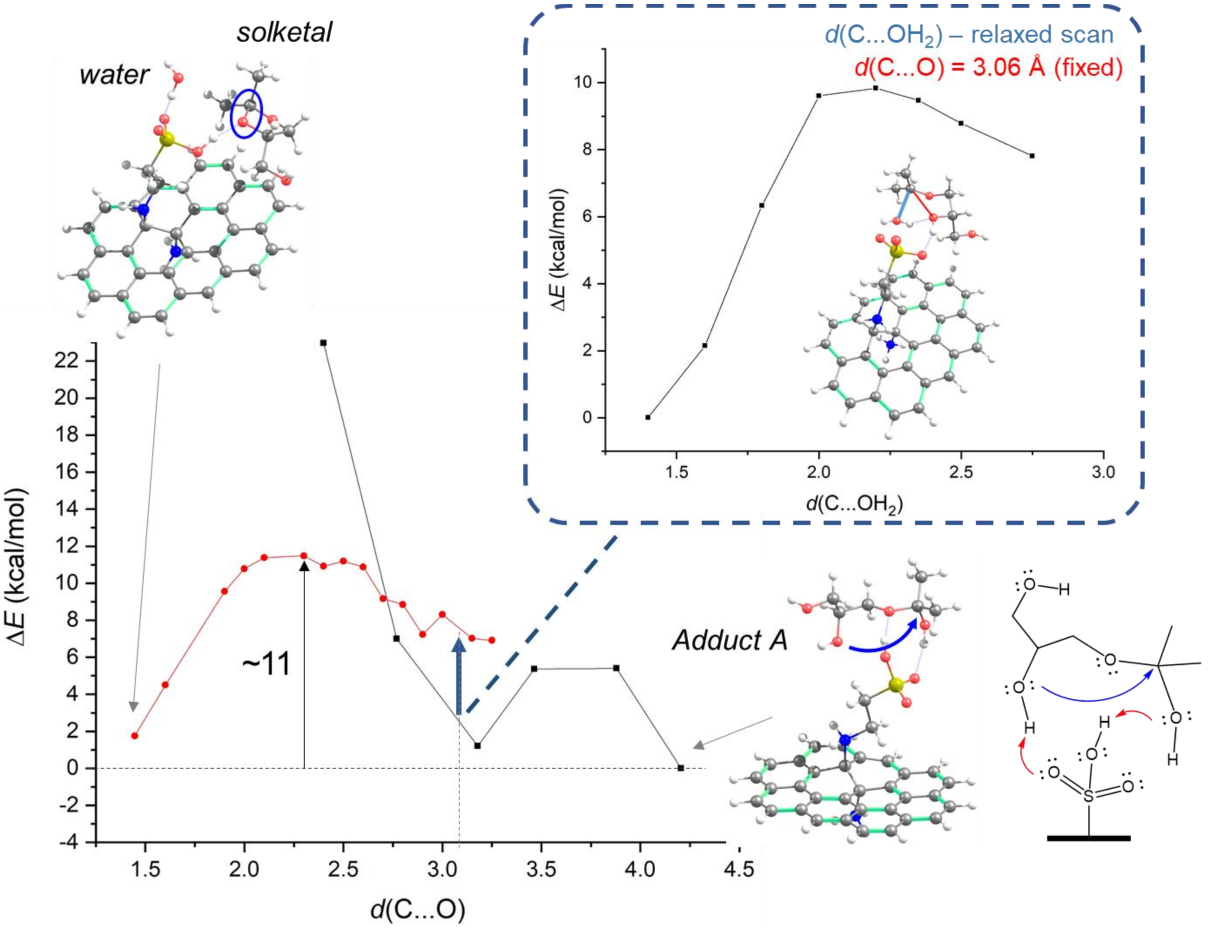

Supplementary Fig. 13. **Cyclization of non-protonated adducts A**. Forward (black; starting from an adduct structure) and reversed (red; starting from the solketal structure) relaxed scans for the cyclization of non-protonated adduct A along the marked C…O coordinate leading to solketal product. A relaxed scan with a fixed C···O distance connecting the forward and backward scans is shown in the inset. ωB97X-D/6-31+G(d)/SMD(solvent=acetone).

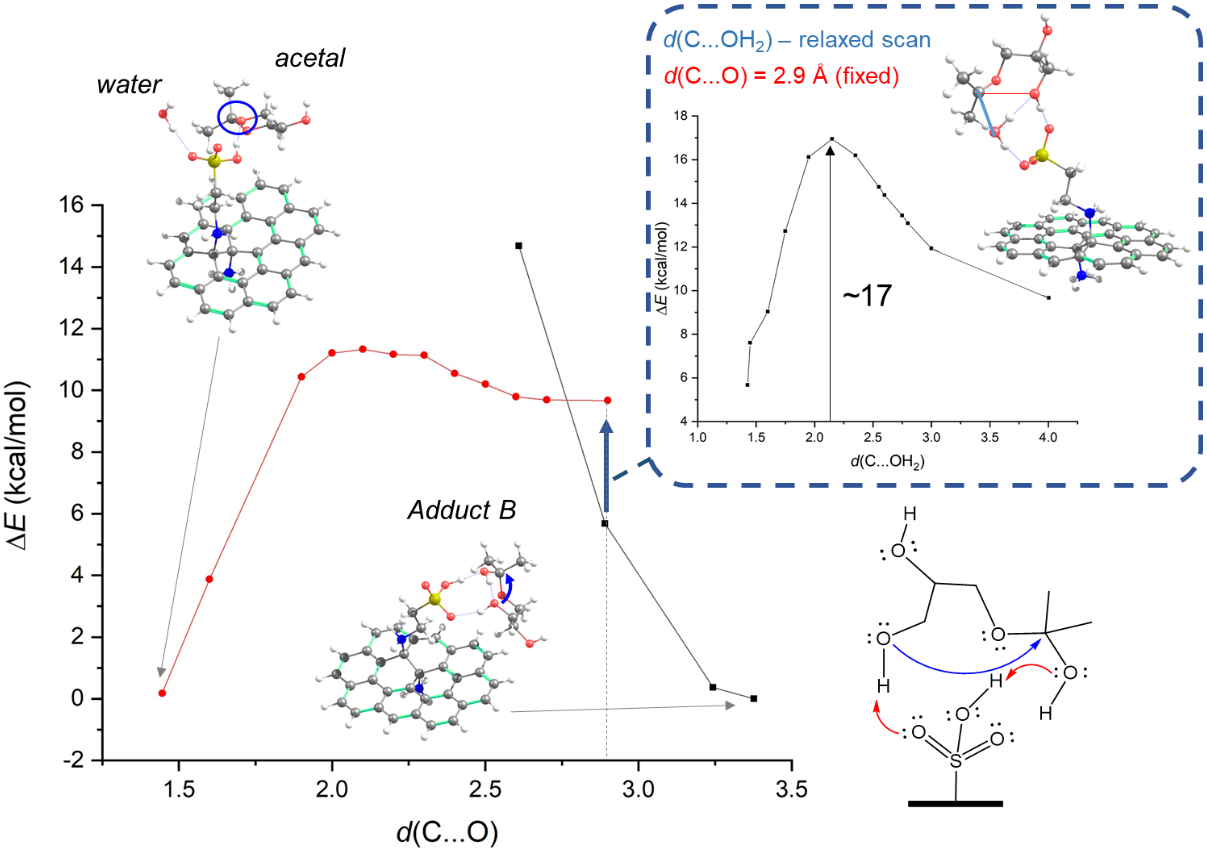

Supplementary Fig. 14. Cyclization of non-protonated adduct B. Forward (black; starting from an adduct structure) and reversed (red; starting from the acetal structure) relaxed scans for the cyclization of non-protonated adduct B along the marked C…O coordinate leading to solketal product. A relaxed scan with a fixed C···O distance connecting the forward and backward scans is shown in the inset. ωB97X-D/6-31+G(d)/SMD(solvent=acetone).

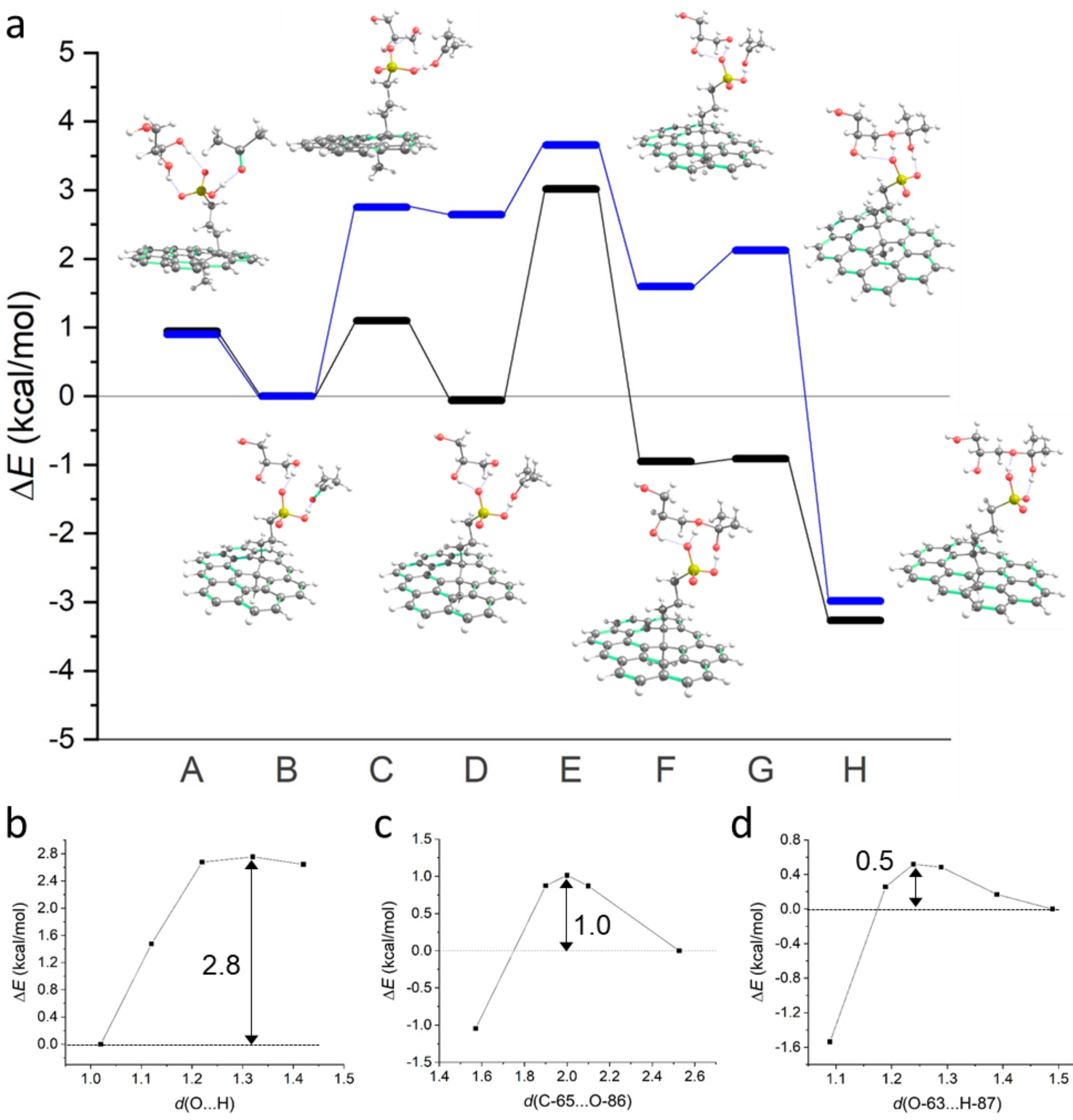

Supplementary Fig. 15. Comparison of G-ASA with G-alkylsulfonic acid. Comparison of the reaction energy profiles (in kcal/mol) of the first phase of the reaction for the G-ASA catalyst (black) and its G-alkylsulfonic analog (blue): formation of adduct A (steps 2a and 3a in Figure 4) along the C(acetone) ···O(gly) coordinate. The structures displayed here represent the G-alkylsulfonic catalyst analog. The corresponding structures of G-ASA are shown in Supplementary Fig. 8. (b) Relaxed scan along the O...H coordinate corresponding to a proton transfer from sulfonic group to an oxygen atom of acetone (step B → C → D in panel a). (c) Relaxed scan along the C-65(acetone)···O-86(gly) coordinate (step D → E → F in panel a). (d) Relaxed scan along O-63···H-87 coordinate; proton transfer from an adduct to the sulfonate group (step F → G → H in panel a). Computational level: ωB97X-D/6-31+G(d)/SMD(solvent=acetone).

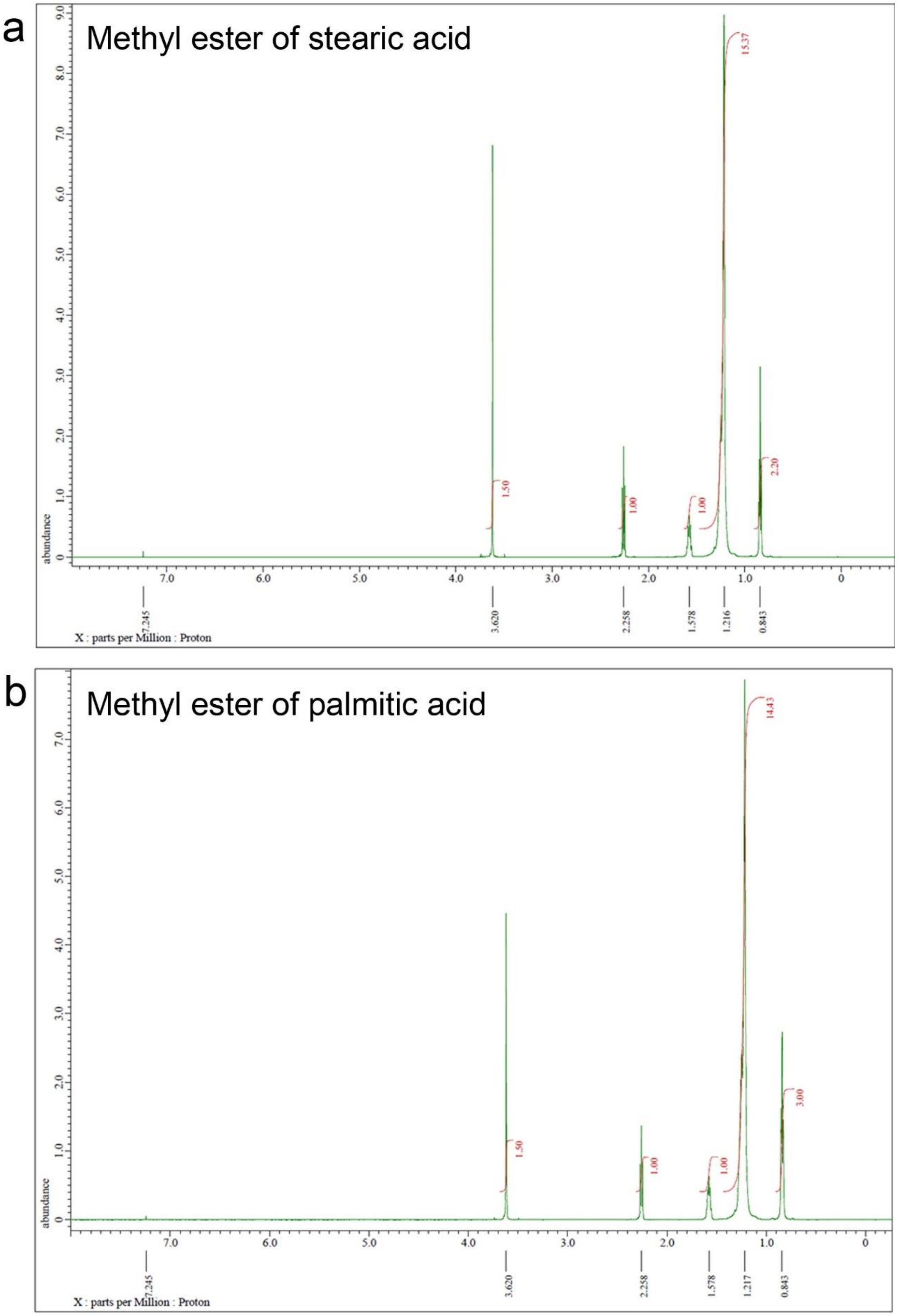

Supplementary Fig. 16. NMR spectrum of fatty acid esters. NMR spectrum of (a) stearic acid and (b) palmitic acid methyl ester

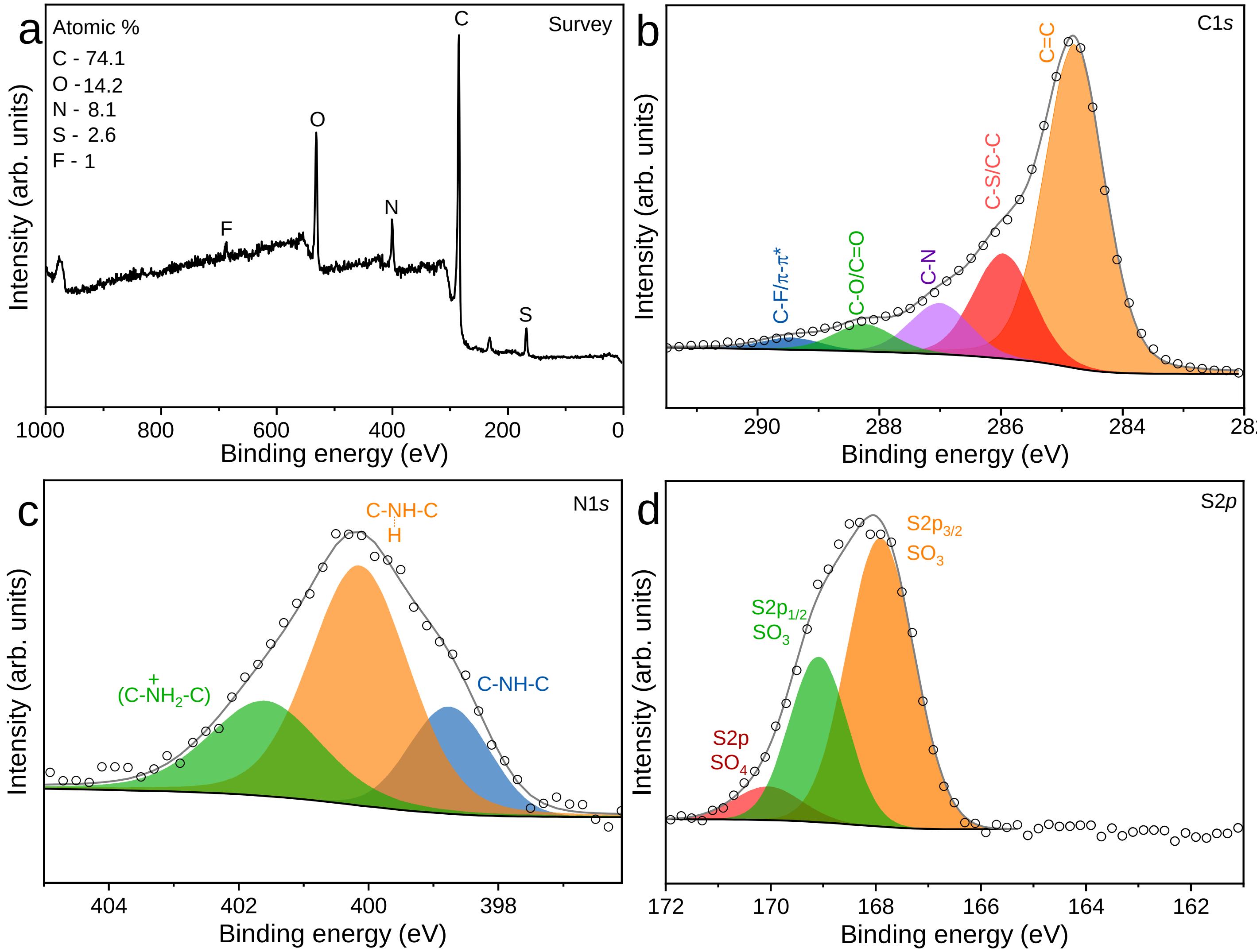

Supplementary Fig. 17. XPS spectrum of G-ASA after 5 esterification reactions. (a) survey, core-level, (b) C 1s, (c) N 1s, and (d) S 2p.

Supplementary References

1 Chai, J. D. & Head-Gordon, M. Long-range corrected hybrid density functionals with damped atom-atom dispersion corrections. *Phys. Chem. Chem. Phys.* **10**, 6615-6620 (2008).

2 Ditchfield, R., Hehre, W. J. & Pople, J. A. Self-consistent molecular-orbital methods. IX. An extended gaussian-type basis for molecular-orbital studies of organic molecules. *J. Chem. Phys.* **54**, 724-728 (1971).

3 Marenich, A. V., Cramer, C. J. & Truhlar, D. G. Universal solvation model based on solute electron density and on a continuum model of the solvent defined by the bulk dielectric constant and atomic surface tensions. *J. Phys. Chem. B* **113**, 6378-6396 (2009).

4 Gaussian 16 Rev. C.01 (Wallingford, CT, 2016).

5 Li, L., Koranyi, T. I., Sels, B. F. & Pescarmona, P. P. Highly-efficient conversion of glycerol to solketal over heterogeneous Lewis acid catalysts. *Green Chem.* **14**, 1611-1619 (2012).

6 Mallesham, B., Sudarsanam, P., Raju, G. & Reddy, B. M. Design of highly efficient Mo and W-promoted SnO_2 solid acids for heterogeneous catalysis: Acetalization of bio-glycerol. *Green Chem.* **15**, 478-489 (2013).

7 Mallesham, B., Sudarsanam, P. & Reddy, B. M. Eco-friendly synthesis of bio-additive fuels from renewable glycerol using nanocrystalline SnO_2-based solid acids. *Catal. Sci. Technol.* **4**, 803-813 (2014).

8 Souza, T. E., Portilho, M. F., Souza, P. M. T. G., Souza, P. P. & Oliveira, L. C. A. Modified niobium oxyhydroxide catalyst: An acetalization reaction to produce bio-additives for sustainable use of waste glycerol. *ChemCatChem* **6**, 2961-2969 (2014).

9 Nair, G. S. *et al.* Glycerol utilization: solvent-free acetalisation over niobia catalysts. *Catal. Sci. Technol.* **2**, 1173-1179 (2012).

10 Ghosh, A. *et al.* A green approach for the preparation of a surfactant embedded sulfonated carbon catalyst towards glycerol acetalization reactions. *Catal. Sci. Technol.* **10**, 4827-4844 (2020).

11 Rodrigues, R., Goncalves, M., Mandelli, D., Pescarmona, P. P. & Carvalho, W. A. Solvent-free conversion of glycerol to solketal catalysed by activated carbons functionalised with acid groups. *Catal. Sci. Technol.* **4**, 2293-2301 (2014).

12 Tayade, K. N., Mishra, M., Munusamy, K. & Somani, R. S. Synthesis of aluminium triflate-grafted MCM-41 as a water-tolerant acid catalyst for the ketalization of glycerol with acetone. *Catal. Sci. Technol.* **5**, 2427-2440 (2015).

13 Vicente, G., Melero, J. A., Morales, G., Paniagua, M. & Martin, E. Acetalisation of bio-glycerol with acetone to produce solketal over sulfonic mesostructured silicas. *Green Chem.* **12**, 899-907 (2010).

14 Laskar, I. B., Rajkumari, K., Gupta, R. & Rokhum, L. Acid-functionalized mesoporous polymer-catalyzed acetalization of glycerol to solketal, a potential fuel additive under solvent-free conditions. *Energ. Fuel* **32**, 12567-12576 (2018).

15 Goncalves, M., Rodrigues, R., Galhardo, T. S. & Carvalho, W. A. Highly selective acetalization of glycerol with acetone to solketal over acidic carbon-based catalysts from biodiesel waste. *Fuel* **181**, 46-54 (2016).

16 Menezes, F. D. L., Guimaraes, M. D. O. & da Silva, M. J. Highly selective $SnCl_2$-catalyzed solketal synthesis at room ternperature. *Ind. Eng. Chem. Res.* **52**, 16709-16713 (2013).

17 Toda, M. *et al.* Green chemistry - Biodiesel made with sugar catalyst. *Nature* **438**, 178-178 (2005).

18 Wang, X. Q. *et al.* Sulfonated ordered mesoporous carbon as a stable and highly active protonic acid catalyst. *Chem. Mater.* **19(10)**, 2395-2397 (2007).

19 Liu, F. J. *et al.* Transesterification catalyzed by ionic liquids on superhydrophobic mesoporous polymers: Heterogeneous catalysts that are faster than homogeneous catalysts. *J. Am. Chem. Soc.* **134**, 16948-16950 (2012).

20 Nakajima, K. & Hara, M. Amorphous carbon with SO_3H groups as a solid bronsted acid catalyst. *ACS Catal.* **2**, 1296-1304 (2012).

21 Zhang, X. M. *et al.* Polystyrene sulphonic acid resins with enhanced acid strength via macromolecular self-assembly within confined nanospace. *Nat. Commun.* **5** (2014).

22 Jia, R., Ren, J. W., Liu, X. H., Lu, G. Z. & Wang, Y. Q. Design and synthesis of sulfonated carbons with amphiphilic properties. *J. Mater. Chem. A* **2(29)**, 11195-11201 (2014).

23 Wang, Y. *et al.* Monodispersed hollow SO_3H-functionalized carbon/silica as efficient solid acid catalyst for esterification of oleic acid. *ACS Appl. Mater. Inter.* **7(48)**, 26767-26775 (2015).
24 Zhang, H. L. *et al.* Highly Efficient Sulfonic/Carboxylic Dual-Acid Synergistic Catalysis for Esterification Enabled by Sulfur-Rich Graphene Oxide. *Chemsuschem* **10**, 3352-3357 (2017).
25 Wang, Y. T. *et al.* Biodiesel production from esterification of oleic acid by a sulfonated magnetic solid acid catalyst. *Renew. Energ.* **139**, 688-695 (2019).
26 Liu, F. S. *et al.* Dilute sulfonic acid post functionalized metal organic framework as a heterogeneous acid catalyst for esterification to produce biodiesel. *Fuel* **266**, 117149 (2020).
