# Supplementary material for: Acidic graphene organocatalyst for the superior transformation of wastes into high-added-value chemicals
Source: Nat Commun. 2023 Mar 13;14:1373. doi: 10.1038/s41467-023-36602-0 (PMC10011376; doi:10.1038/s41467-023-36602-0)
Supplement: Supplementary file 3 — Source Data [file 41467_2023_36602_MOESM3_ESM.zip › Solketal synthesis over GR-SO3H.pptx]

## Slide 1
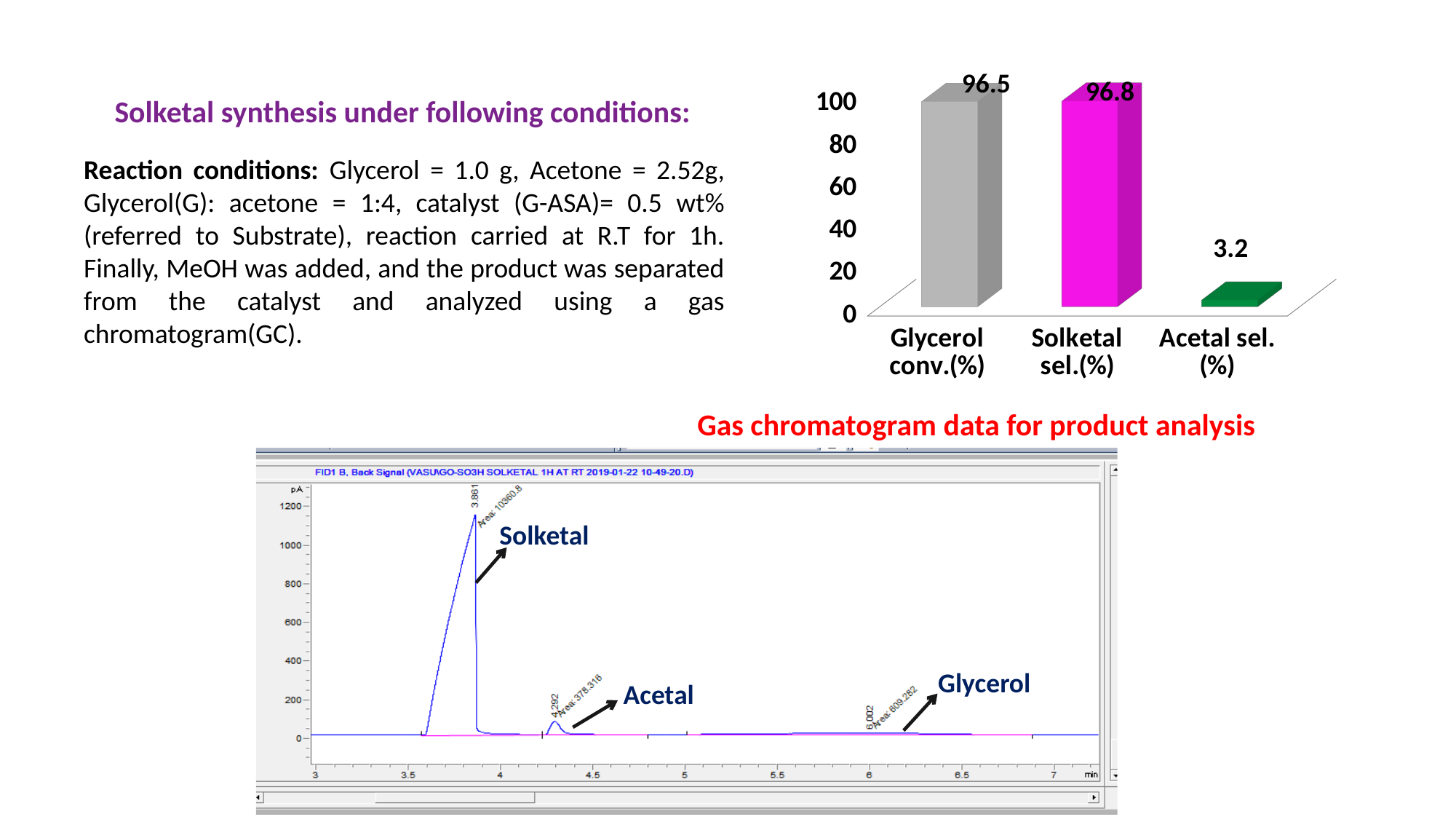

96.5
[unsupported chart]
96.8
3.2
Solketal synthesis under following conditions:
Reaction conditions: Glycerol = 1.0 g, Acetone = 2.52g, Glycerol(G): acetone = 1:4, catalyst (G-ASA)= 0.5 wt% (referred to Substrate), reaction carried at R.T for 1h. Finally, MeOH was added, and the product was separated from the catalyst and analyzed using a gas chromatogram(GC).
Gas chromatogram data for product analysis
Solketal
Glycerol
Acetal

## Slide 2
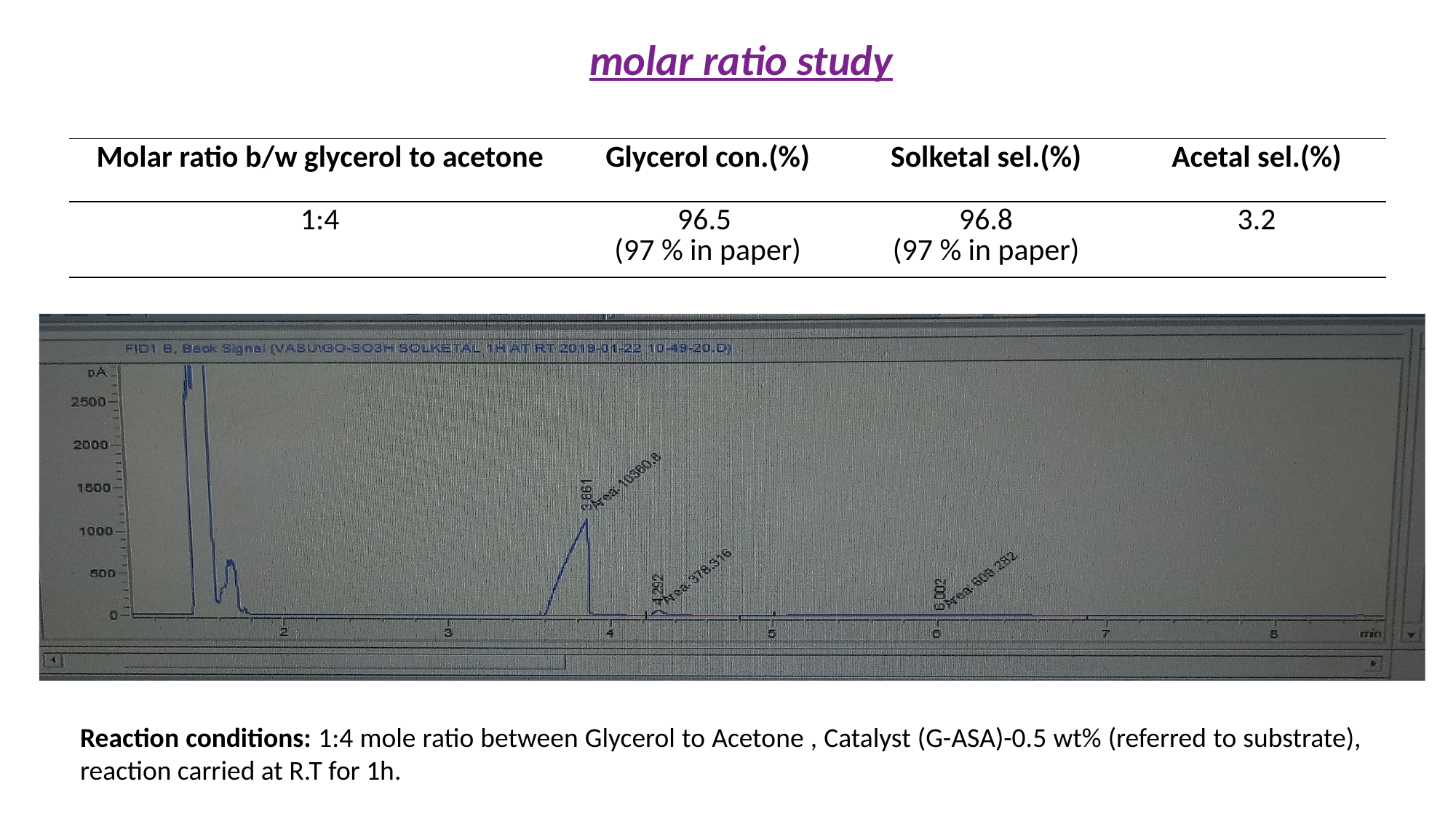

molar ratio study
| Molar ratio b/w glycerol to acetone | Glycerol con.(%) | Solketal sel.(%) | Acetal sel.(%) |
| --- | --- | --- | --- |
| 1:4 | 96.5 (97 % in paper) | 96.8 (97 % in paper) | 3.2 |
Reaction conditions: 1:4 mole ratio between Glycerol to Acetone , Catalyst (G-ASA)-0.5 wt% (referred to substrate), reaction carried at R.T for 1h.

## Slide 3
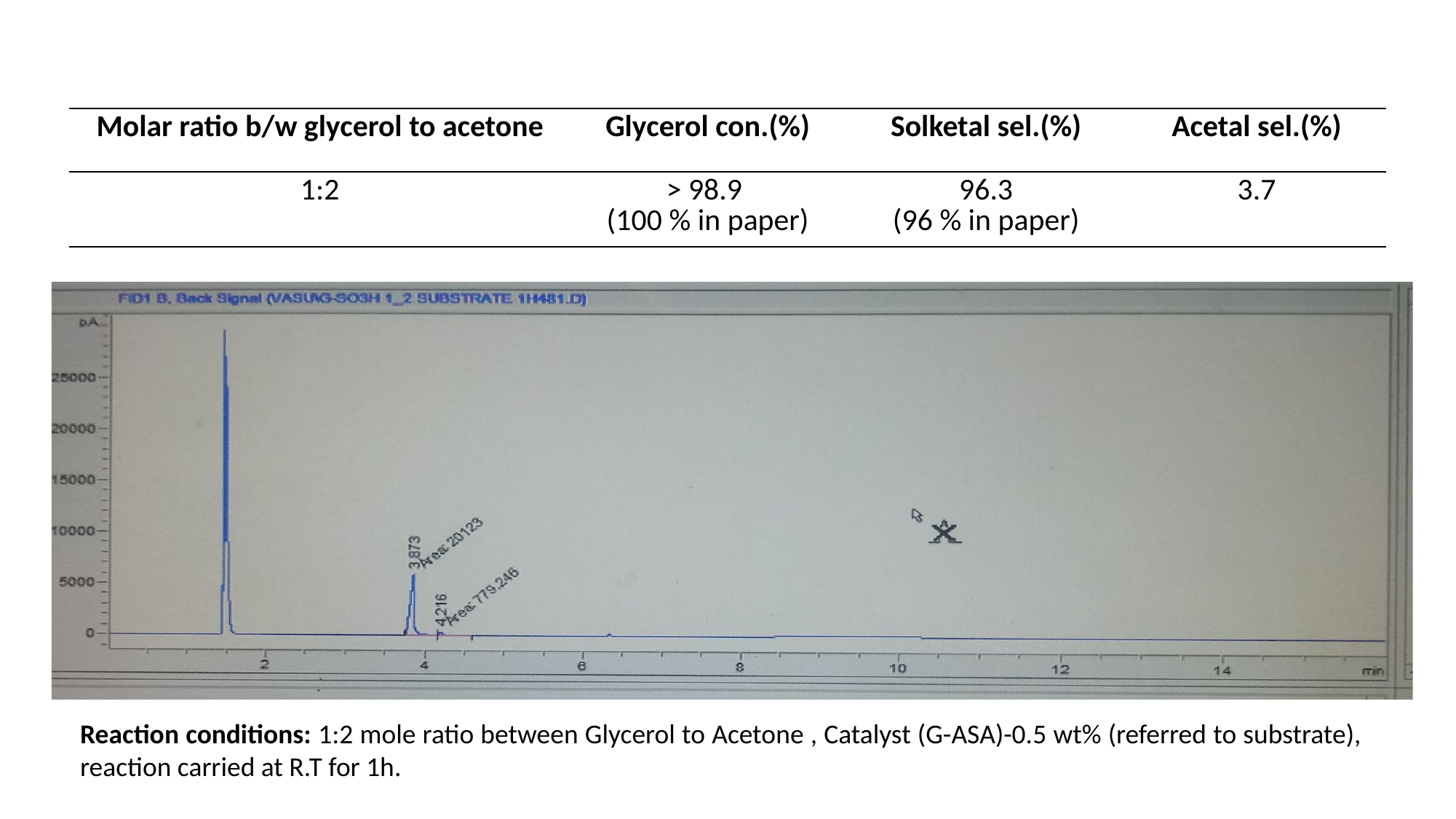

| Molar ratio b/w glycerol to acetone | Glycerol con.(%) | Solketal sel.(%) | Acetal sel.(%) |
| --- | --- | --- | --- |
| 1:2 | > 98.9 (100 % in paper) | 96.3 (96 % in paper) | 3.7 |
Reaction conditions: 1:2 mole ratio between Glycerol to Acetone , Catalyst (G-ASA)-0.5 wt% (referred to substrate), reaction carried at R.T for 1h.

## Slide 4
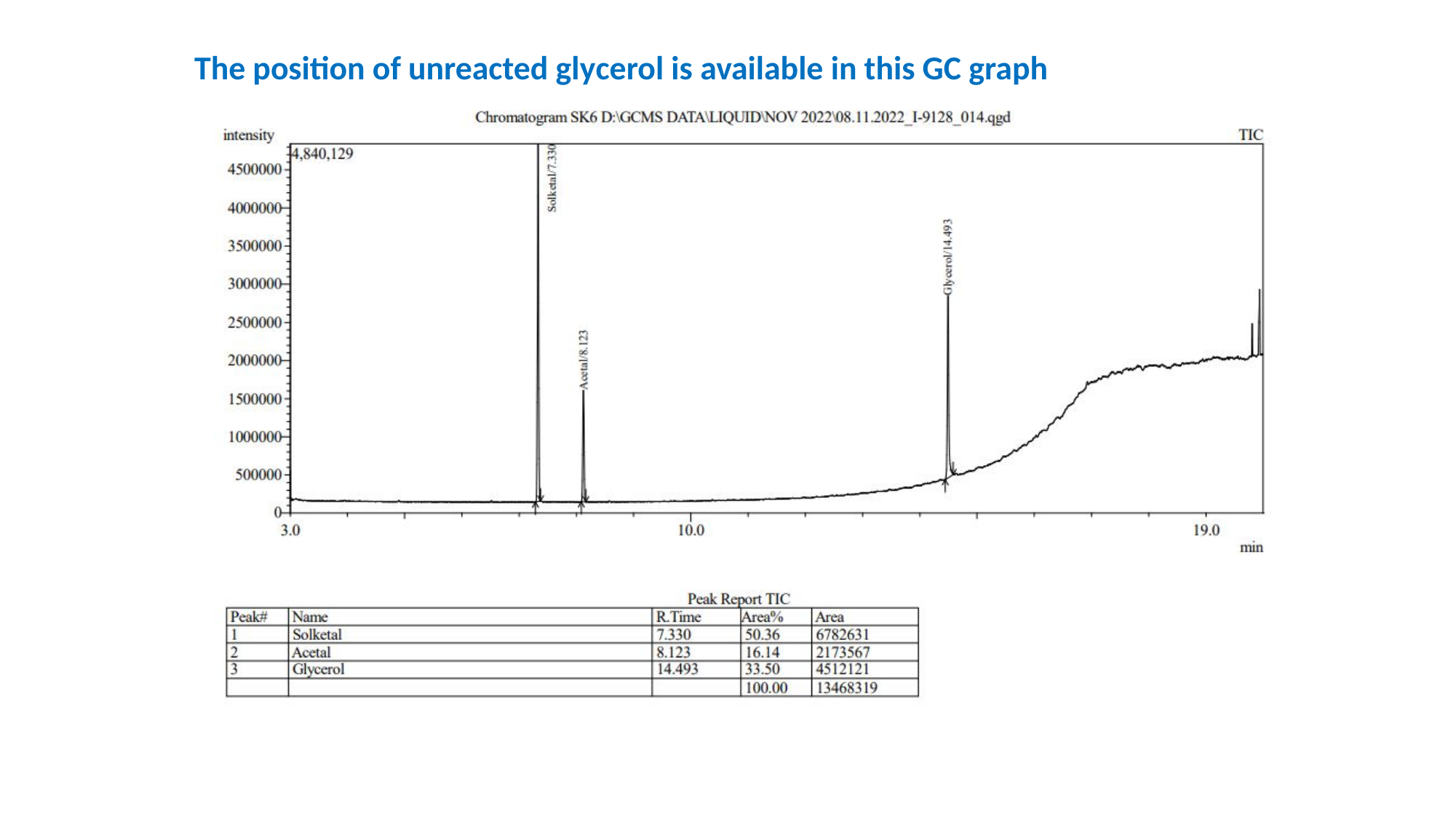

The position of unreacted glycerol is available in this GC graph
